# Patient and Strain Characteristics Associated With *Clostridium difficile* Transmission and Adverse Outcomes

**DOI:** 10.1093/cid/ciy302

**Published:** 2018-04-12

**Authors:** Jessica S H Martin, David W Eyre, Warren N Fawley, David Griffiths, Kerrie Davies, Damian P C Mawer, Timothy E A Peto, Derrick W Crook, A Sarah Walker, Mark H Wilcox

**Affiliations:** 1 Leeds Teaching Hospitals & University of Leeds, United Kingdom; 2 Nuffield Department of Medicine, United Kingdom; 3 NIHR Oxford Biomedical Research Centre, University of Oxford, United Kingdom; 4 Public Health England–Leeds Regional Laboratory, United Kingdom; 5 Public Health England, Colindale, United Kingdom

**Keywords:** *Clostridium difficile*, whole-genome sequencing, outcome

## Abstract

**Background:**

No study has used whole-genome sequencing (WGS) to investigate risk factors for Clostridium difficile (CD) transmission between cases, or assessed the impact of recent acquisition on patient outcome.

**Methods:**

This 20 month retrospective cohort study included consecutive cytotoxin-positive diarrheal samples, which underwent culture, ribotyping, and WGS (Illumina). Sequenced isolates were compared using single nucleotide variants (SNVs). Independent predictors of acquisition from another case, onward transmission, 120-day recurrence, and 30-day mortality were identified using logistic regression with backwards elimination.

**Results:**

Of 660 CD cases, 640 (97%) were sequenced, of which 567 (89%) shared a ribotype with a prior case, but only 227 (35%) were ≤2 SNVs from a prior case, supporting recent acquisition. Plausible (<2 SNVs) recent ward-based acquisition from a symptomatic case was more frequent in certain ribotypes; 64% (67/105) for ribotype-027 cases, compared with 11% (6/57) for ribotype-078. Independent risk factors (adjusted *P* < .05) for CD acquisition included older age, longer inpatient duration, and ribotype; these factors, and male sex, increased onward transmission. Patients with a plausible donor had a greater risk of recurrence (adjusted *P* = .001) and trended towards greater 30-day mortality (adjusted *P* = .06). Ribotype had no additional mortality or recurrence impact after adjusting for acquisition (*P* > .1).

**Conclusions:**

Greater transmission of certain lineages suggests CD may have different reservoirs and modes of transmission. Acquiring CD from a recent case is associated with poorer clinical outcomes. Clinical characteristics associated with increased healthcare-associated CD transmission could be used to target preventative interventions.

## Teaser

Combining epidemiological, clinical, and genomic data for *Clostridium difficile* cases offers fresh insight into characteristics associated with transmission and outcome. *C. difficile* lineages may have different reservoirs and modes of transmission, and recent strain acquisition is associated with poorer outcomes.

*Clostridium difficile* (renamed *Clostridioides difficile*) infection (CDI) is the most common hospital-associated infection in the United States [1] and challenges healthcare providers worldwide. CDI is associated with increased all-cause 30-day mortality, and particular strain types, for example BI/NAP1/ribotype-027, have been associated with worse clinical outcomes [2, 3].

The temporal and spatial epidemiology of *C. difficile* strains is frequently monitored using ribotyping [4] in Europe [5] and pulsed-field gel electrophoresis [6] and ribotyping in North America. Whole genome sequencing (WGS) is a highly discriminatory fingerprinting technique that has been used to demonstrate the inter-continental spread of recently evolved clones (BI/NAP1/ribotype-027) [7], and to determine strain transmission between CDI cases [8–10]. Previous epidemiological studies have shown that typically only a third of new CDI cases are acquired from a symptomatic donor [9, 11, 12], but no study has compared the clinical attributes and strain types of cases with and without healthcare-associated acquisition, as defined by WGS.

The aim of this study was to find out whether using a WGS-based analysis of *C. difficile* strains, rather than less-discriminatory techniques such as ribotyping, reveals insights into the patient and strain characteristics most closely associated with CDI transmission and outcome. We investigated *C. difficile* transmission between symptomatic cases within a UK region, diagnosed via a reference method (cell cytotoxicity), using both ribotyping and WGS. We determined patient and strain risk factors for both transmitting the infection onwards and acquiring *C. difficile* from a prior symptomatic case. We assessed the clinical consequences of case-to-case transmission by investigating the likelihood of CDI recurrence and death in patients with/without a probable donor.

## METHODS

Leeds Teaching Hospitals’ NHS Trust’s laboratory provides diagnostic services for all inpatient and community care in the metropolitan area of Leeds, UK (population 750000). It comprises 3 large teaching hospitals and numerous smaller community care providers. During this study, Leeds Teaching Hospitals’ infection control policy stipulated that any patient with ≥1 episode of unexplained diarrhea should be isolated and tested for CDI [13]. Patients were source-isolated from the day of onset of diarrhea to being either >48 hours symptom-free or discharged from hospital. Standard CDI treatment during this period was oral metronidazole for non-severe cases and oral vancomycin for severe cases (total white cell count >15.0x10^9^/L, serum creatinine >50% above baseline, or evidence of colitis).

Consecutive diarrheal samples submitted for CDI testing and confirmed as toxin-positive by a cell cytotoxicity assay were cultured from a single colony, ribotyped [4], and sequenced using Illumina technology [9]; the samples included any repeat positives >7 days later from the same patient. Samples were compared using ribotype and differences in single nucleotide variants (SNVs) obtained from maximum likelihood phylogenies (Supplementary Methods). Sequences generated during this study can be found on the NCBI short read archive under BioProject PRJNA317528 (http://www.ncbi.nlm.nih.gov/bioproject/PRJNA317528).

CDI cases were defined as the first culture-positive *C. difficile* sample from each cytotoxin-positive patient, plus repeat culture-positive samples >10 SNVs distinct from all prior samples (extremely unlikely to arise through mutation during the study) [8, 9, 14]. For each case, data were collected on sex; age; all inpatient episodes in the 6 months before and after each sample, including all ward-stays, day-case procedures, dialysis, and chemotherapy attendances; time between stool sampling and source isolation; home postcode district; general practice location; severity biomarkers at diagnosis (summarized as severity score 0–3; one point each for age >65 years, peak creatinine >176μmol/L, and peripheral blood white cell count >20000 cells/µL) [15]; recurrence (occurring in the next 120 days); and all-cause 30-day mortality. Recurrent infection was defined as a repeat cytotoxin-positive sample >14 days after the first positive sample (reliable information was not available about symptom resolution between samples). Consensus definitions were used for community-associated (CA), indeterminate (I), community-onset healthcare facility–associated (CO-HCFA), and hospital-onset healthcare facility–associated (HO-HCFA) CDI (Supplementary Figure S1) [3].

For each new sequenced case (n = 640), we considered all previous case strains (including repeat positives within individuals) as potential transmission sources. Using rates of *C. difficile* evolution and within-host diversity, direct transmission was considered plausible where a prior “donor” isolate was within 0–2 SNVs of a “recipient” [9]. Cases within 3–10 SNVs were likely to share a common source in the last 5 years, but direct transmission was unlikely. Cases >10 SNVs from all previous cases were considered genetically distinct and unlikely to share a common source with another case during the study.

### Statistical Methods

Univariable associations between these factors and cases having acquired *C. difficile* from a previous, closely genetically-related case or having transmitted CDI onwards (≤2 SNV threshold) were investigated using Kruskal-Wallis/rank-sum (continuous factors) and chi-squared/exact (categorical factors) tests. For multivariable logistic models for acquisition, transmission, 120-day recurrence, and 30-day mortality, severity score (missing in n = 124 [19%] where blood tests were not done) and source isolation at onset (missing in n = 269 [42%]; 150 pre–January 2011, when this was not collected, and 119 post–January 2011, due to poor documentation) were imputed multiply (50 times) using both chained estimating equations [16] (Stata version 14.1; including all factors above and all four outcome variables) and boxcox-transforming continuous variables for normality. Independent predictors were selected using backwards elimination (exit *P* > .1 to fit an exploratory model) using fractional polynomials to investigate non-linearity [17]. Independent effects of each ribotype with >25 cases (vs all other ribotypes) were considered for inclusion in main models. Sensitivity analyses included ribotype as an 8-level factor (7 common vs others combined), together with all factors identified as independently prognostic in any model or all other factors.

The study was approved by the Berkshire Research Ethics Committee (10/H0505/83) and the Health Research Authority (8-05[e]2010).

## RESULTS

From 1 August 2010 to 24 April 2012, of 16873 tested diarrheal samples from hospital and community patients in the Leeds region, 888 (5%) were *C. difficile* cytotoxin-positive, representing 660 CDI cases in 625 patients (excluding repeats ≤10 SNVs). A total of 831 *C. difficile* isolates were successfully sequenced, representing 640/660 (97%) cases (Supplementary Figure S2).

Median age at diagnosis was 76 years (inter-quartile range [IQR] 62–84), and 389 (59%) cases were female patients. The incidence of healthcare-associated CDI was 4.2 HO-HCFA and CO-HCFA infections per 10000 overnight stays. The incidence of community-associated CDI was 143 CA and I infections per 100000 Leeds community per annum. Incidence declined during the study period (Supplementary Figure S3). Thirty-day all-cause mortality was 19% (124/660) for all cases. The most frequent ribotypes were 027 (multi-locus sequence type, ST1; 106 [16%] cases), 015 (ST10/ST44/ST45; 64 [10%]), and 078 (ST11; 58 [9%]). The rate of identification of genetically distinct strains (>10 SNVs from previous cases) was approximately constant during the study (Supplementary Figure S4).

### CDI Recurrence

Sequential samples were analyzed for CDI cases with ≥120 days of follow up (1 August 2010 to 26 December 2011). Removing repeat samples ≤10 SNVs within 14 days of the first sample, 114/539 (21%) first CDI cases in this period had a recurrence. Of these, 95 (83% of recurrences, 18% of first CDI cases) were within 0–2 SNVs (ie, probable relapse) and 16 (14% of recurrences, 3% of first CDI cases) were >10 SNVs (ie, probable re-infection). The duration between the first sample and recurrence was a median 26 days (IQR 22–40; range 15–103) for 0–2 SNVs repeats, and 32 days (IQR 27–65; range 14–93) for >10 SNVs repeats (*P* = .06). The remaining 3 recurrences were 3 SNVs distant, occurring 24–91 days following the first sample. Of the 114 first CDI cases with recurrences, 98 (86% of recurrences, 18% of first cases) had recurrent infection with the same ribotype; 1 patient had reinfection with a genetically distinct (1340 SNVs distant) *C. difficile* strain of the same ribotype.

### *C. difficile* Transmission From Prior Cases

Out of the new cases, 567 (89%) had the same ribotype as at least one previous CDI in the study population, but only 227 (35%) were 0–2 SNVs from a previous isolate, suggesting recent acquisition from the symptomatic population, while 286 (45%) were >10 SNVs from all prior isolates (Table 1). These proportions were similar when restricting to cases from January 2011 onwards (175/449 [39%] 0–2 SNVs and 176/449 [39%] >10 SNVs, *P* = .20), demonstrating there was no significant difference in the proportion of cases with a recent donor when accounting for the potentially reduced pool of donors in the first months of the study.

**Table 1.** Genetic Relatedness of 640 New Clostridium difficile Infection Cases to Any Previous Isolate

Type of Relationship/Contact	Same Ribotype	0 SNV	0–2 SNV	3–10 SNV	0–10 SNV
Relationship to all previous study isolates at this threshold	567 (89%)	162 (25%)	227 (35%)	127 (20%)	354 (55%)
Genetically linked to any previous case on the same ward at the same time	164 (26%)	91 (14%)	115 (18%)	9 (1%)	124 (19%)
Within plausible infectious and incubation periods^a^	139 (22%)	84 (13%)	105 (16%)	6 (1%)	111 (17%)
Genetically linked to any previous case on the same ward at a different time	144 (23%)	20 (3%)	30 (5%)	34 (5%)	64 (10%)
Within 4 wk	54 (8%)	13 (2%)	16 (3%)	9 (1%)	25 (4%)
Plausible community contact	83 (13%)	8 (1%)	13 (2%)	8 (1%)	21 (3%)
Shared general practice	27 (4%)	4 (1%)	10 (2%)	1 (0%)	11 (2%)
Postcode district	77 (12%)	8 (1%)	11 (2%)	9 (1%)	20 (3%)
Genetically related but no contact of any kind identified	176 (28%)	43 (7%)	69 (11%)	76 (12%)	145 (23%)
CA/I	67 (10%)	12 (2%)	21 (3%)	32 (5%)	53 (8%)

Prior contact considered in order shown (same ward same time (direct), same ward different time (spore), community) with the exception of any spore contamination lasting <28 days which was considered more plausible than direct transmission outside 56-day donor infectious period and 90-day recipient incubation periods.

Abbreviations: CA, community associated; CDI, *Clostridium difficile* infection; I, indeterminate; SNV, single nucleotide variants.

^a^Fifty-six–day donor infectious period and 90-day recipient incubation [18].

Only 115 (18%) new CDI cases had any prior direct ward contact (same ward, same day) with a 0–2 SNV donor; this contact was for a median of 8 days (IQR 4–16; range 1–59), with a median of 20 days (IQR 6–45; range 1–191) between the donor’s most recent positive and the recipient’s first positive sample (counting the most recent donor if multiple donors were identified). Other potential transmission routes are summarized in Table 1.

### Impact of Acquisition on Clinical Outcomes

All-cause 30-day mortality was 27% (62/227) for patients with a previous potential donor, but only 12% (34/286) for those with genetically distinct strains (*P* < .001). Recurrence (>14 days after first test) was also more frequent in cases with any potential donor (29%, 65/227) versus those without (16%, 47/286; *P* = .001). The association between acquisition and mortality did not persist after adjusting for other independent predictors (Table 2; *P* = .06), but there was an independent association between acquisition and recurrence (*P* < .001). Importantly, other than ribotype-020 marginally increasing the risk of recurrence vs all other ribotypes combined (*P* = .06), there was no additional impact of ribotype on either mortality or recurrence after adjusting for the (stronger) impact of acquisition itself (*P* > .1); thus, any excess risk of adverse outcomes associated univariably with specific lineages (ribotype-027 in particular, with univariable *P* < .001 for associations with mortality and recurrence) was mediated through acquisition from a previous symptomatic donor.

**Table 2.** Results of a Multivariate Model to Identify Independent Predictors of Acquiring Clostridium difficile Infection From a Previous Case, Onwards Transmission, Recurrence, or 30-day Mortality

	30-d Mortality	Recurrence	Recipients	Potential Donors
	Yes vs No	Yes vs No	With Previous 0–2 SNV Donor vs all Prior Samples >2 SNVs	0–2 SNV Potential Donor to Any Subsequent Case vs All Subsequent Samples >2 SNVs
Factor	OR (95% CI) *P*	OR (95% CI) *P*	OR (95% CI) *P*	OR (95% CI) *P*
Age (per 10 y older)	1.41 (1.21, 1.64) *P* < .001	–	1.32 (1.16, 1.51) *P* < .001	1.22 (1.08, 1.36) *P* = .001
Female vs male	0.63 (0.41, 0.97) *P* = .04	1.68 (1.12, 2.54) *P* = .01	–	0.66 (0.43, 1.01) *P* = .06
Time from start of study to sample (per month longer)	–	–	^a^	0.91 (0.88, 0.95) *P* < .001
Inpatient days (<12 wk) pre-diagnosis (per week longer)	1.07 (1.00, 1.14) *P* = .04	–	1.19 (1.12, 1.27) *P* < .001	1.11 (1.05, 1.18) *P* < .001
Origin: CA	1.00	1.00	–	–
I vs CA	0.94 (0.27, 3.28) *P* = .93	0.77 (0.29, 2.08) *P* = .61	–	–
CO-HCFA vs CA	1.68 (0.71, 3.99) *P* = .24	1.75 (0.93, 3.28) *P* = .08	–	–
HO-HCFA vs CA	2.25 (1.04, 5.87) *P* = .04	1.24 (0.71, 2.18) *P* = .44	–	–
Ribotype 027	–	^b^	179.6 (59.6, 541.0) *P* < .001	80.7 (34.1, 190.8) *P* < .001
Ribotype 015	–	^b^	2.07 (0.94, 4.54) *P* = .07	^b^
Ribotype 078	–	^b^	6.84 (3.38, 13.8) *P* < .001	5.89 (3.04, 11.3) *P* < .001
Ribotype 014	–	^b^	3.40 (1.59, 7.25) *P* = .002	3.86 (1.91, 7.81) *P* < .001
Ribotype 020	–	1.98 (0.96, 4.09) *P* = .06	4.13 (1.78, 9.56) *P* = .001	3.89 (1.76, 8.59) *P* = .001
Ribotype 002	–	^b^	2.05 (0.95, 4.43) *P* = .07	2.15 (1.05, 4.43) *P* = .04
Ribotype 001/072	–	^b^	4.93 (2.02, 12.0) *P* < .001	4.38 (1.85, 10.4) *P* = .001
Other ribotype	–	1.00	1.00	1.00
Having a previous donor 0–2 SNVs vs >10 SNVs	1.62 (0.98, 2.66) *P* = .06	2.07 (1.33, 3.20) *P* = .001	N/A	N/A
Having a previous donor 0–2 SNV vs 3–10 SNVs vs >10 SNVs	1.63 (0.90, 2.96) *P* = .11	1.15 (0.66, 1.99) *P* = .63	N/A	N/A

"–" means factor not selected in final model. N/A means this factor not applicable for this model (either is the outcome, occurs after the outcome or is a competing outcome). See Supplementary Tables S1 and S2 for sensitivity analysis including all 8 ribotype categories.

Abbreviations: CA, community associated: CI, confidence interval; CDI, *Clostridium difficile* infection; CO-HCFA, community-onset healthcare facility–associated; HO-HCFA, hospital-onset healthcare facility–associated; I, indeterminate; OR, odds ratio; SNV, single nucleotide variants.

^a^Included in final model with the best fitting transform: inverse of months from study start, with lower odds of identifying a previous 0–2 SNV donor lower only in the first month of the study (OR = 0.42 per 1/(months from start of study) higher (95% CI 0.21, 0.85; *P* = .02).

^b^Included with “other ribotype” in final model; no evidence of difference vs other ribotypes (*P* > .1).

### Risk Factors for Acquiring CDI From a Previous Case

The factors most strongly univariably associated with having a previous potential donor (0–2 SNVs) were older age, origin of infection (CA/I/CO-HCFA/HO-HCFA), more inpatient days in the 12 weeks pre-diagnosis, higher severity score, and ribotype (Table 3). More recent hospital exposures were associated with greater proportions of CDIs closely related to a prior case (Supplementary Figure S5, Supplementary Table S3), both at SNV thresholds consistent with direct and recent indirect transmission (suggesting these cases arose from a reduced hospital reservoir of strains). The range of ribotypes causing CDI was broad regardless of healthcare or community origin of infection, although ribotype-027 was particularly associated with close healthcare exposure (*P* = .02 vs other ribotypes combined; Supplementary Figure S6).

**Table 3.** Characteristics of Genetically-matched and Distinct Potential Recipients

Factor	All New Cases (N = 640)	New Cases With Previous 0–2 SNV Donor (N = 227 [35%])	New Cases With Previous 3–10 SNV Donor (N = 127 [20%])	New Cases With All Prior Samples >10 SNVs (N = 286 [45%])	*P* Value 0–2 vs 3–10 vs >10 SNVs	*P* Value 0–2 vs >2 SNVs
**Predictors**						
Female	376 (59%)	121 (53%)	73 (57%)	182 (64%)	*P* = .06	*P* = .04
Age, years	76 (61–84)	81 (70–87)	71 (58–82)	71 (51–82)	*P* < .001	*P* < .001
Study start to first sample, days	255 (126–404)	250 (157–398)	326 (161–396)	234 (82–422)	*P* = .04	*P* = .30
Type of CDI						
CA	121 (19%)	26 (11%)	28 (22%)	67 (23%)	*P* = .001 (see Supplementary Figure S5)	*P* < .001
I	46 (7%)	11 (5%)	10 (8%)	25 (9%)		
CO-HCFA	126 (20%)	42 (19%)	29 (23%)	55 (19%)		
HO-HCFA	347 (54%)	148 (65%)	60 (47%)	139 (49%)		
Inpatient on day of diagnosis	511 (80%)	184 (81%)	102 (80%)	225 (79%)	*P* = .79	*P* = .57
Inpatient days in the 12 wk pre-diagnosis	16 (4–40)	27 (10–48)	13 (3–28)	13 (2–34)	*P* < .001	*P* < .001
Source isolation on the day of symptom onset^a^	177/246 (72%)	78/111 (70%)	36/50 (72%)	63/85 (74%)	*P* = .84	*P* = .60
Ribotype (if >25 cases)						
027	105 (16%)	99 (44%)	3 (2%)	3 (1%)	*P* < .001	*P* < .001
015	62 (10%)	12 (5%)	23 (18%)	27 (9%)	*P* < .001	*P* = .005
078	57 (9%)	26 (11%)	28 (22%)	3 (1%)	*P* < .001	*P* = .09
002	55 (9%)	15 (7%)	13 (10%)	27 (9%)	*P* = .40	*P* = .18
014	49 (8%)	18 (8%)	2 (2%)	29 (10%)	*P* = .004	*P* = .85
020	38 (6%)	14 (6%)	11 (9%)	13 (5%)	*P* = .26	*P* = .86
001/072	29 (5%)	13 (6%)	4 (3%)	12 (4%)	*P* = .56	*P* = .32
Other	245 (38%)	30 (13%)	43 (34%)	172 (60%)	*P* < .001	*P* < .001
**Outcomes**						
*Clostridium difficile* severity score (n = 516)						
0	119 (23%)	20 (11%)	28 (26%)	71 (31%)	*P* < .001	*P* < .001
1	268 (52%)	111 (61%)	51 (48%)	106 (47%)		
2	112 (22%)	43 (23%)	24 (22%)	45 (20%)		
3	17 (3%)	9 (5%)	4 (4%)	4 (2%)		
Inpatient days in the 12 wk post-diagnosis	16 (4–38)	20 (5–42)	12 (4–30)	15 (4–34)	*P* = .12	*P* = .05
>1 positive sample	158 (25%)	73 (32%)	30 (24%)	55 (19%)	*P* = .003	*P* = .001
Recurrent CDI (≥14 d)	136 (21%)	65 (29%)	24 (19%)	47 (16%)	*P* = .001	*P* = .003
30-d mortality	120 (19%)	62 (27%)	24 (19%)	34 (12%)	*P* < .001	*P* < .001

Numbers show N (%) or median (inter-quartile range), and univariable Kruskal-Wallis (continuous variable) or chi-squared (categorical) tests. Fisher’s exact tests were used where any cell percentage was <5% or any frequency was <5. For ribotypes each comparison is that specific ribotype vs all others pooled.

Abbreviations: CA, community associated: CI, confidence interval; CDI, *Clostridium difficile* infection; CO-HCFA, community-onset healthcare facility–associated; HO-HCFA, hospital-onset healthcare facility–associated; I, indeterminate; OR, odds ratio; SNV, single nucleotide variants.

^a^Applicable only to hospital-onset cases: collected from January 2011 where data was available in clinical documentation.

**Figure 1.**
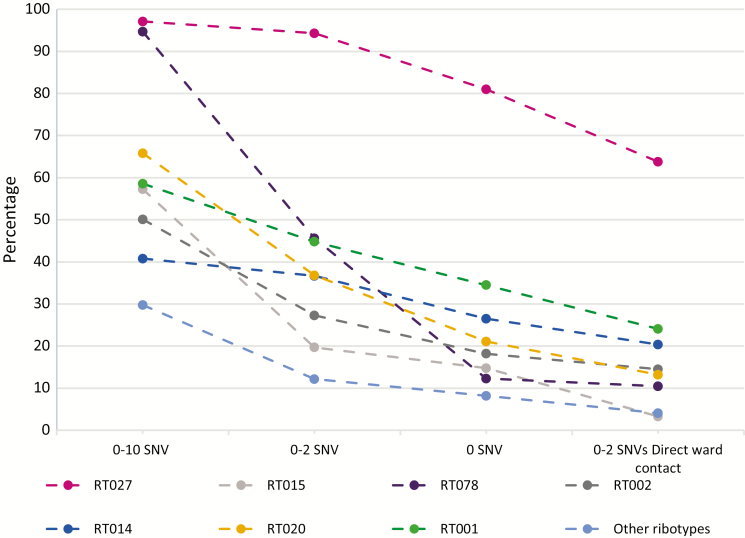
Genetic relatedness to previous Clostridium difficile infection cases by ribotype (if >25 cases). Abbreviation: SNV, single nucleotide variants.

The proportion of cases with potential donors at differing levels of genetic relatedness varied markedly according to CDI ribotype (Figure 1, Supplementary Table S4). The vast majority of ribotype-027 (102/105 [97%]) and ribotype-078 (54/57 [95%]) CDIs had a previous case within 0–10 SNVs; however, genetically close matches (0–2 SNV) occurred in 94% (99/105) and 46% (26/57), respectively. Furthermore, 64% (67/105) of ribotype-027 but only 11% (6/57) of ribotype-078 cases had shared time on a ward with a potential donor ≤2 SNV, supporting greater rates of hospital-based acquisition for ribotype-027 compared with ribotype-078. Other common ribotypes (e.g. 015 and 002) were much more genetically diverse; thus, lower proportions of these CDIs had prior strains within 0–10 SNVs (56% [35/62] ribotype-015 and 51% [28/55] ribotype-002), 0–2 SNVs (19% [12/62] and 27% [15/55], respectively), and, particularly, 0–2 SNVs with ward contact (3% [2/62] and 15% [8/55], respectively).

In a multivariable analysis, the odds of acquiring CDI from a previous symptomatic donor (0–2 SNVs) remained independently higher for specific ribotypes (particularly 027; to a lesser extent, 078/014/020/001/072), older individuals, and those with more inpatient days in the preceding 12 weeks; as expected, risk was lower in the first study month, where fewer previous cases had been sequenced (Table 2). There was no independent effect of origin of infection (CA/I/CO-HCFA/HO-HCFA) after adjusting for these factors (*P* = .71), which was plausibly better explained by total inpatient days in the last 12 weeks rather than time since last exposure, categorized as per surveillance criteria.

### Risk Factors for Onward Transmission

A total of 228 (36%) CDI cases were potential donors (0–2 SNVs) to ≥1 subsequent cases. The factors most strongly univariably associated with being a potential donor were older age, male sex, earlier diagnosis in the study period, more inpatient days during the 12 weeks before diagnosis, higher severity score, and ribotype (Table 4). In 20/115 (17%) recipients, the most plausible transmission event (using the most recent donor if multiple donors were identified) followed a donor’s second or third diarrheal sample, a median of 49 days (IQR 21–95; range 8–129) after the initial test. Overall, 45/115 (39%) of the most plausible donors had recurrent infections.

**Table 4.** Characteristics of Genetically-matched and Distinct Potential Donors

Factor	All New Cases	0–2 SNV Potential Donors to Any Subsequent Case^a^	Subsequent Case 3–10 SNV	All Subsequent Cases >10 SNV	*P* Value 0–2 vs 3–10 vs >10 SNVs	*P* Value 0–2 vs >2 SNVs
	N = 640	N = 228 (36%)	N = 131 (20%)	N = 281 (44%)		
**Predictors**						
Female	376 (59%)	117 (51%)	78 (60%)	181 (64%)	*P* = .01	*P* = .004
Age, years	76 (61–84)	80 (70–87)	74 (58–83)	71 (53–83)	*P* < .001	*P* < .001
Days from start of study to sample	255 (126–404)	224 (112–342)	255 (111–374)	335 (158–495)	*P* < .001	*P* < .001
Type of CDI						
CA	121 (19%)	27 (12%)	36 (27%)	58 (21%)	*P* < .001	*P* = .002
I	46 (7%)	13 (6%)	9 (7%)	24 (9%)		
CO-HCFA	126 (20%)	37 (16%)	29 (22%)	60 (21%)		
HO-HCFA	347 (54%)	151 (66%)	57 (44%)	139 (49%)		
Inpatient on day of diagnosis	511 (80%)	193 (85%)	100 (76%)	218 (78%)	*P* = .08	*P* = .02
Inpatient days in the 12 wk pre-diagnosis	16 (4–40)	21 (9–45)	10 (1–26)	14 (3–38)	*P* < .001	*P* < .001
Source isolation on the day of symptom onset^b^	177/246 (72%)	67/100 (67%)	29/39 (74%)	81/107 (76%)	*P* = .36	*P* = .15
Ribotype (if >25 cases)						
027	105 (16%)	98 (43%)	4 (3%)	3 (1%)	*P* < .001	*P* < .001
015	62 (10%)	12 (5%)	24 (18%)	26 (9%)	*P* < .001	*P* = .005
078	57 (9%)	26 (11%)	28 (21%)	3 (1%)	*P* < .001	*P* = .10
002	55 (9%)	16 (7%)	17 (13%)	22 (8%)	*P* = .13	*P* = .29
014	49 (8%)	19 (8%)	2 (2%)	28 (10%)	*P* = .01	*P* = .63
020	38 (6%)	15 (7%)	9 (7%)	14 (5%)	*P* = .66	*P* = .61
001/072	29 (5%)	13 (6%)	4 (3%)	12 (4%)	*P* = .53	*P* = .32
Other	245 (38%)	29 (13%)	43 (33%)	173 (62%)	*P* < .001	*P* < .001
**Outcomes**						
*Clostridium difficile* severity score (N = 516 cases)						
0	119 (23%)	18 (10%)	27 (26%)	74 (33%)	*P* < .001	*P* < .001
1	268 (52%)	110 (59%)	52 (50%)	106 (47%)		
2	112 (22%)	48 (26%)	24 (23%)	40 (18%)		
3	17 (3%)	10 (5%)	2 (2%)	5 (2%)		
Inpatient days in the 12 wk post-diagnosis	16 (4–38)	19 (7–41)	16 (3–41)	13 (3–33)	*P* = .04	*P* = .01
>1 positive sample	158 (25%)	73 (32%)	32 (24%)	53 (19%)	*P* = .003	*P* = .001
Recurrent CDI (≥14 d)	136 (21%)	60 (26%)	28 (21%)	48 (17%)	*P* = .04	*P* = .02
30-d mortality	120 (19%)	56 (25%)	18 (14%)	46 (16%)	*P* = .02	*P* = .005

Numbers show N (%) or median (IQR), and univariable Kruskal-Wallis (continuous variable) or chi-squared (categorical) tests. Fisher’s exact tests were used where any cell percentage was <5% or any frequency was <5. For ribotypes each comparison is that specific ribotype vs all others pooled.

Abbreviations: CA, community associated: CDI, *Clostridium difficile* infection; CO-HCFA, community-onset healthcare facility–associated; HO-HCFA, hospital-onset healthcare facility–associated; I, indeterminate; SNV, single nucleotide variants.

^a^including any case with a subsequent case within the 0–2 SNV threshold as a potential donor as the specific transmission route is unknown.

^b^Applicable only to hospital-onset cases: collected from January 2011 where data was available in clinical documentation.

In a multivariable analysis, the odds of onwards transmission to a new symptomatic CDI case (0–2 SNVs) remained independently higher for specific ribotypes (particularly 027; to a lesser extent, 078/014/020/002/001/072), older individuals, men, and those with more inpatient days in the preceding 12 weeks; as expected, odds decreased slightly over the study period (Table 2). After adjusting for these factors, there was no independent effect of donor origin of infection (*P* = .93) or inpatient days post-diagnosis (*P* = .47).

## CONCLUSIONS

Our study identified risk factors connected with healthcare-associated transmission of CDI, and measured the clinical consequences of recent *C. difficile* acquisition from a symptomatic case, having robustly defined genetic relatedness between cases using WGS. We examined a large cohort of cases using a sensitive approach to sampling and diagnosis based on toxin testing, which has been shown to have a high predictive value for true CDI [19, 20]. By combining WGS and conventional ribotyping, we found that genetic relatedness of strains to previous cases varies significantly according to ribotype, suggesting *C. difficile* lineages may have different reservoirs and modes of transmission. Interestingly, however, we did not demonstrate an independent relationship between ribotype and adverse outcome, as some previous studies have shown; recurrence and mortality were more strongly associated with strain acquisition from a recent case than with strain type.

We found that predictors of recent strain acquisition and onward transmission included more inpatient days during the 12 weeks before diagnosis, older age, and certain ribotypes. These data could help to identify a high-risk population for healthcare-associated strain acquisition that could be used when planning future preemptive *C. difficile* interventions, such as vaccination [21] or prophylaxis [22]. Interestingly, the total number of days spent in hospital in the 12 weeks prior to infection was more closely associated with strain acquisition than conventional community vs. hospital definitions [3].

Our results provide new evidence that certain *C. difficile* ribotypes have higher levels of in-hospital transmissibility. Specifically, ribotype-027 strains were predominantly clonal, with approximately two-thirds of ribotype-027 CDIs having recent ward contact with a genetically matched case. This epidemic ribotype has been associated with more frequent poor outcomes and is known to be a recently evolved clone [7, 23]. Conversely, ribotype-078, a strain also associated with poor outcomes [24, 25], was largely clonal, but only 11% of ribotype-078 CDIs occurred in patients with recent ward contact with a genetically matched case. Other authors have also shown low patient-to-patient transmission for this strain [26]. *C. difficile* ribotype-078 is commonly found in livestock [27, 28], hence food and farm exposure are potential alternative environmental sources [29–31]. Notably, other common disease-causing lineages (eg, 015, 002) demonstrated few genetic matches, so were unlikely to have been recently acquired from a common source.

We show that 30-day mortality and 120-day disease recurrence were each more likely in cases where CDI was plausibly acquired from a recent donor. The association between recent acquisition and recurrence persisted after adjusting for other important factors, including age and ribotype; hence, this association was not due to confounding by ribotype-027 in particular. The association between recent acquisition and outcome may reflect a host contribution (eg, multiple comorbidities) predisposing a patient to both the acquisition and the poor outcome. Nevertheless, these findings strongly support the importance of infection control interventions in targeting symptomatic CDI cases to reduce the risk of transmission to vulnerable patients, and so prevent particularly poor outcomes in these individuals. Furthermore, in an era where nosocomial CDI incidence is increasingly viewed as a healthcare quality indicator [32], our data suggest that evidence demonstrating control of in-hospital *C. difficile* transmission (ie, lack of strain-relatedness between cases) could be used as a specific measure of prevention and control effectiveness.

Our study has several limitations. In particular, it is impossible to confirm patient-to-patient *C. difficile* transmission retrospectively, even with detailed epidemiological data and WGS. The few sequence failures may have led to donor/recipient genetic relationships being missed. Transmission events outside the geographical boundaries of Leeds could also have been missed. We did not examine asymptomatic carriers and, therefore, our results apply only to transmission leading from and to disease; there is growing evidence that asymptomatic carriers may play a key role in wider *C. difficile* transmission [12, 33, 34]. However, as WGS-matched samples from the entire study period were considered as possible donors, the influence of clinically significant (but unknown) intermediate carriers has been accounted for, as two cases related by a common source were still linked to each other in the study. Furthermore, a low threshold for patient sampling and automatic *C. difficile* testing of diarrheal specimens will have enhanced our detection of possible *C. difficile* donors; such UK-recommended practice has been associated with a marked decrease in CDI incidents [5, 35]. The incidence of ribotype-027 has decreased in the UK since the study period; the proportion of cases with a donor (or recipient) may be lower if the study is repeated. Data for some potentially influential factors were incomplete or not available (including proton-pump inhibitor use, antibiotic use, and co-morbidities), and so we have not considered these in our main analyses. Understanding the possible role of antibiotic use in transmission/acquisition of *C. difficile* is complex, not least given heterogeneity of exposure (including polypharmacy). Interestingly, antibiotic use in the 7 or 90 days before diagnosis was not strongly associated with either transmission or acquisition from a previous symptomatic donor (both *P* > .2; Supplementary Table S5) in patients for whom these data were available. Lastly, data were not available on diarrhea severity or duration; however, CDI severity criteria and repeat tests were used as proxies for these.

In summary, WGS and detailed epidemiological data demonstrate that acquisition of a *C. difficile* strain that caused a previous symptomatic case is associated with poorer clinical outcome. Targeting preemptive interventions to patients at high risk of being donors or recipients will likely further reduce *C. difficile* transmission and infection rates within healthcare institutions. This will help to improve patient experiences and outcomes. Notably, we also demonstrated variable healthcare-associated transmission of *C. difficile* strain types, suggesting CDI is not a homogeneous entity, but is likely to have different reservoirs and modes of transmission.

## Supplementary Data

Supplementary materials are available at *Clinical Infectious Diseases* online. Consisting of data provided by the authors to benefit the reader, the posted materials are not copyedited and are the sole responsibility of the authors, so questions or comments should be addressed to the corresponding author.

Supplementary Materials

## Notes

D. W. C. and T. E. A. P. are NIHR senior investigators. J. S. H. M. is an NIHR academic clinical fellow and D. W. E. is a NIHR clinical lecturer.

***Disclaimer.*** Funders had no role in study design, data collection, analysis, or writing of the report.

***Financial support.*** This work was supported by the UK Clinical Research Collaboration through the Wellcome Trust (grant 087646/Z/08/Z), the Medical Research Council (grant G0800778), and the National Institute for Health Research; and by the NIHR Oxford Biomedical Research Centre.

***Potential conflicts of interest.*** K. D. received grants to her institution from Astellas Pharma Europe Ltd, Alere, bioMerieux, Cepheid, Pfizer, Sanofi-Pasteur, and Summit. M. H. W. reports grants and personal fees from Actelion, Cubist, Astellas, Merck, Sanofi-Pasteur, Summit, Seres, Biomerieux, Qiagen, and Pfizer; grants from Da Volterra; personal fees from Valneva, Ferring, and Synthetic Biologics; personal fees and other from Alere; other from Astellas Pharma; and non-financial support from Astellas Pharma during the conduct of the study. M. H. W. also reports personal fees from Astra Zeneca, Nabriva, Pfizer, Roche, The Medicine Company, Basilea, Bayer, Allergan, Menarini, Motif Biosciences, AiCuris, Antabio, and Spero; and grants and personal fees from Abbott, European Tissue Symposium, Paratek, Tetraphase, and Surface Skins outside the submitted work. All authors have submitted the ICMJE Form for Disclosure of Potential Conflicts of Interest. Conflicts that the editors consider relevant to the content of the manuscript have been disclosed.
