# Supplementary material for: Patient and Strain Characteristics Associated With Clostridium difficile Transmission and Adverse Outcomes
Source: Clin Infect Dis. 2018 Apr 12;67(9):1379–87. doi: 10.1093/cid/ciy302 (PMC6186849; doi:10.1093/cid/ciy302)
Supplement: Supplementary Materials [file ciy302_suppl_supplementary_material.docx]

**Supplementary material**

1. Supplementary methods: whole-genome sequencing methods and analysis
2. Figure S1: Surveillance criteria for the origin of *C. difficile* infection
3. Figure S2: Samples and cases 01 August 2010 to 24 April 2012.
4. Figure S3: Incidence of all new CDI cases, overall and by origin
5. Figure S4: Introduction of genetically distinct (>10 SNV) strains during study period (01 August 2010 -24 April 2012).
6. Table S1: Independent predictors of acquiring CDI from a previous case, onwards transmission, recurrence or 30-day mortality adjusting for all factors identified as prognostic in any model
7. Table S2: Independent predictors of acquiring CDI from a previous case, onwards transmission, recurrence or 30-day mortality: adjusting for all factors in Tables 3 and 4 (main text)
8. Figure S5: Genetic relatedness of new CDI cases (n=640) to any previous isolate based on origin of recipient’s infection.
9. Table S3: Table to show genetic relatedness of new CDI cases to any previous isolate based on origin of recipient’s infection.
10. Figure S6. (a) Ribotype diversity and (b) Simpson’s index of diversity by origin of infection
11. Table S4: Genetic relatedness to previous CDI cases by ribotype (if >25 cases)
12. Table S5:
    1. Antibiotic use in genetically-matched and distinct recipients
    2. Antibiotic use in genetically-matched and distinct potential donors
13. References

**Supplementary methods: whole-genome sequencing methods and analysis.**

DNA was extracted using a commercial kit (FastDNA, MP Biomedicals, California, USA; QIAamp, Qiagen, Hilden, Germany; QuickGene, Fujifilm, Tokyo, Japan), from a single colony sub-cultured onto a Columbia blood agar plate and incubated for 48 h. A combination of standard Illumina and adapted protocols was used to produce multiplexed paired-end libraries. Briefly, indexed pools of 96 samples were sequenced at the Wellcome Trust Centre for Human Genetics, Oxford, UK, using the Illumina HiSeq 2000 platform (Illumina Inc, San Diego, CA) generating 100 base pair paired-end reads. Reads were mapped with Stampy^1^ (without Burrows-Wheeler Aligner pre-mapping, using an expected substitution rate of 0.01) to the *C. difficile* reference genome 630 (Genbank: AM180355.1), masking annotated mobile genetic elements. Single nucleotide variants (SNVs) identified with Samtools^2^ mpileup with the extended base-alignment quality flag. A consensus of ≥75% was required to support a SNV, and calls were required to be homozygous under a diploid model. Only SNVs supported by ≥5 reads, including one in each direction were accepted. Multi-locus sequence types (STs) were inferred *in silico.* Sequences were excluded if <70% of the reference genome sites were called.

Sequences were compared using maximum likelihood phylogenies. To improve computational efficiency in identifying closely related sequences, sequences within ≤100 SNVs of any other sequence were initially pooled into groups. All variable sites within each group were then identified, sequences with <70% of these variable sites called (either as wild-type or variant) were excluded from further analysis, as these represent possible cross-contamination amongst closely related samples in the same sequencing batch. Within each group, variable sites that were not called (either as wild type or variant) in ≥70% of samples were also excluded from further analysis, as such sites may represent either regions of the genome that are difficult to identify with certainty from short-read sequencing or regions that are variably present or absent.

For each group of sequences within ≤100 SNVs, maximum likelihood phylogenetic trees were constructed using PhyML 3.0^3^, using a generalised time reversible substitution model, and the “BEST” tree topology search operation option. Pairwise SNV differences for closely related samples were obtained from these phylogenetic trees. Observed pairwise SNV differences were used for differences >100 SNVs.

One sample was excluded as <70% of the reference genome was identified and 5 were excluded as <70% of the identified variable sites were identified. In the remaining sequences, laboratory determined ribotypes were compared with *in silico* STs. In 10 samples discordant results were obtained, and these samples were also therefore excluded from the analysis. In the remaining 831 sequences a median (IQR) [range] 84.5% (83.9-85.1%) [74.3-90.2%] of the reference genome was called as variant / wild-type.

**Figure S1: Surveillance criteria for the origin of *C. difficile* infection.^4^**

**
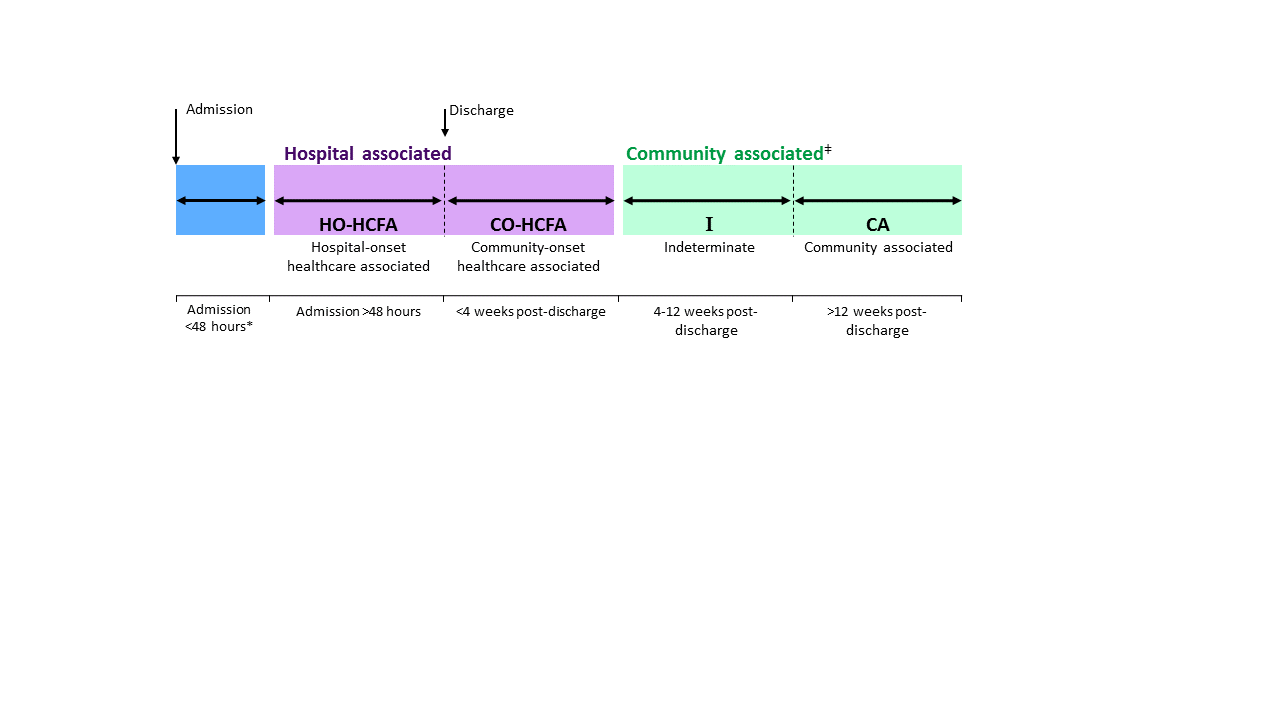
**

* Patients developing CDI within the first 48 hours of admission should have their origin of infection determined by previous hospital admissions.

^ǂ^ In this study, residential/nursing home status was considered to be a community location (i.e. not healthcare).

**Figure S2: Samples and cases 01 August 2010 to 24 April 2012.**

36 (4%) culture negative

852 successfully cultured and ribotyped (96%)

888 CTA positive samples (5%),

Leeds *C. difficile* testing 01 August 2010 to 24 April 2012

16,873 samples received for cell culture cytotoxin assay (CTA)

21 (2%) sequence failures or repeats

831(98%) samples with WGS representing 640 CDI cases

(distinct patients and sequence >10 SNV from previous samples)

15,985 CTA negative (95%)

**Figure S3: Incidence of all new CDI cases, overall and by origin**

**Figure S4: Introduction of genetically distinct (>10 SNV) strains during study period (01 August 2010 -24 April 2012).**

The first case of each genetic subtype (>10 SNVs from all other isolates) is plotted on a new horizontal line, with subsequent cases of the same subtype on the same line. Each case is represented by a dot.

**Table S1 Independent predictors of acquiring CDI from a previous case, onwards transmission, recurrence or 30-day mortality adjusting for all factors identified as prognostic in any model**

|  | **30-day mortality** | **Recurrence** | **Recipients** | **Potential donors** |
| --- | --- | --- | --- | --- |
|  | Yes vs no | Yes vs no | With previous 0-2 SNV donor vs all prior samples >2 SNVs | 0-2 SNV potential donor to any subsequent case vs all subsequent samples >2 SNVs |
| Factor | OR (95% CI) p | OR (95% CI) p | OR (95% CI) p | OR (95% CI) p |
| Age (per 10 years older) | 1.42 (1.21,1.66) p<0.001 | 1.03 (0.93,1.14) p=0.56 | 1.34 (1.18,1.54) p<0.001 | 1.21 (1.08,1.36) p=0.001 |
| Female vs male | 0.63 (0.41,0.97) p=0.04 | 1.70 (1.12,2.59) p=0.01 | 0.76 (0.48,1.20) p=0.24 | 0.66 (0.43,1.03) p=0.07 |
| Time from start of study to sample (per month longer) | 1.02 (0.99,1.06) p=0.23 | 1.00 (0.96,1.03) p=0.84 | * | 0.91 (0.87,0.94) p<0.001 |
| Inpatient days (<12 weeks) pre-diagnosis (per week longer) | 1.08 (1.00,1.14) p=0.05 | 1.00 (0.94,1.07) p=0.95 | 1.20 (1.12,1.29) p<0.001 | 1.11 (1.04,1.19) p=0.003 |
| I vs CA | 0.92 (0.26,3.24) p=0.90 | 0.76 (0.28,2.09) p=0.60 | 0.66 (0.22,1.99) p=0.46 | 0.93 (0.35,2.44) p=0.88 |
| CO-HCFA vs CA | 1.68 (0.70,5.01) p=0.25 | 1.81 (0.92,3.53) p=0.08 | 1.15 (0.53,2.52) p=0.72 | 0.94 (0.44,2.03) p=0.88 |
| HO-HCFA vs CA | 2.36 (1.08,5.15) p=0.03 | 1.23 (0.64,2.37) p=0.53 | 0.84 (0.41,1.73) p=0.64 | 1.13 (0.57,2.22) p=0.73 |
| Ribotype 027 | 0.97 (0.46,2.01) p=0.92 | 1.39 (0.69,2.80) p=0.35 | 177.4 (58.6,536.8) p<0.001 | 98.4 (40.3,240.4) p<0.001 |
| Ribotype 015 | 0.83 (0.34,2.00) p=0.68 | 1.23 (0.59,2.58) p=0.58 | 2.07 (0.94,4.55) p=0.07 | 2.32 (1.07,5.03) p=0.03 |
| Ribotype 078 | 0.91 (0.40,2.08) p=0.82 | 1.75 (0.81,3.74) p=0.15 | 6.64 (3.27,13.5) p<0.001 | 7.09 (3.53,14.2) p<0.001 |
| Ribotype 014 | 0.46 (0.17,1.25) p=0.13 | 0.72 (0.29,1.76) p=0.47 | 3.46 (1.61,7.42) p=0.001 | 4.61 (2.20,9.66) p<0.001 |
| Ribotype 020 | 1.01 (0.40,2.51) p=0.99 | 2.32 (1.05,5.15) p=0.04 | 3.81 (1.62,8.94) p=0.002 | 4.80 (2.09,11.0) p<0.001 |
| Ribotype 002 | 1.13 (0.52,2.45) p=0.76 | 1.03 (0.47,2.25) p=0.94 | 2.00 (0.91,4.36) p=0.08 | 2.60 (1.23,5.52) p=0.01 |
| Ribotype 001/072 | 1.16 (0.42,3.22) p=0.78 | 1.75 (0.71,4.30) p=0.22 | 5.23 (2.13,12.8) p<0.001 | 5.25 (2.14,12.9) p<0.001 |
| Other ribotype | 1.00 | 1.00 | 1.00 | 1.00 |
| Having a previous donor 0-2 SNVs vs >10 SNVs | 1.61 (0.86,2.98) p=0.13 | 1.62 (0.91,2.88) p=0.10 | N/A | N/A |
| Having a previous donor 0-2 SNV vs 3-10 SNVs vs >10 SNVs | 1.56 (0.83,2.94) p=0.17 | 0.96 (0.53,1.74) p=0.89 | N/A | N/A |

* Included in final model with the best fitting transform the inverse of months from study start, with lower odds of identifying a previous 0-2SNV donor lower only in the first 0-1 month of the study (OR=0.42 per 1/(months from start of study) higher (95% CI 0.21,0.86) p=0.02).

Note: OR=odds ratio. Included factors are those selected for any of the four models based on backwards elimination, see Methods. N/A means this factor not applicable for this model (either is the outcome, occurs after the outcome or is a competing outcome). **Table S2 Independent predictors of acquiring CDI from a previous case, onwards transmission, recurrence or 30-day mortality: adjusting for all factors in Tables 3 and 4 (main text)**

|  | 30-day mortality | Recurrence | Recipients | Potential donors |
| --- | --- | --- | --- | --- |
|  | Yes vs no | Yes vs no | With previous 0-2 SNV donor vs all prior samples >2 SNVs | 0-2 SNV potential donor to any subsequent case vs all subsequent samples >2 SNVs |
| Factor | OR (95% CI) p | OR (95% CI) p | OR (95% CI) p | OR (95% CI) p |
| Age (per 10 years older) | 1.41 (1.21,1.65) p<0.001 | 1.03 (0.93,1.76) p=0.57 | 1.33 (1.17,1.53) p<0.001 | 1.15 (1.00,1.33) p=0.05 |
| Female vs male | 0.63 (0.41,0.97 p=0.04 | 1.71 (1.13,2.60) p=0.01 | 0.76 (0.47,1.20) p=0.24 | 0.66 (0.42,1.04) p=0.07 |
| Time from start of study to sample (per month longer) | 1.02 (0.98,1.06) p=0.28 | 1.00 (0.96,1.03) p=0.81 | * | 0.91 (0.87,0.95) p<0.001 |
| Inpatient days (<12 weeks) pre-diagnosis (per week longer) | 1.06 (0.99,1.14) p=0.08 | 1.00 (0.94,1.08) p=0.96 | 1.20 (1.11,1.29) p<0.001 | 1.11 (1.03,1.19) p=0.004 |
| Inpatient days (<12 weeks) post-diagnosis (per week longer) | N/A | N/A | 1.02 (0.95,1.09) p=0.65 | 0.99 (0.92,1.06) p=0.74 |
| Not an inpatient at diagnosis | 1.02 (0.46,2.25) p=0.96 | 1.10 (0.58,2.06) p=0.77 | 1.43 (0.67,3.03) p=0.35 | 0.66 (0.31,1.39) p=0.27 |
| No source isolation at onset | 0.75 (0.37,1.53) p=0.43 | 1.00 (0.48,2.08) p=1.00 | 0.83 (0.41,1.67) p=0.60 | 1.13 (0.55,2.33) p=0.74 |
| I vs CA | 0.91 (0.26,3.20) p=0.88 | 0.77 (0.28,2.12) p=0.61 | 0.62 (0.20,1.90) p=0.40 | 0.85 (0.32,2.27) p=0.75 |
| CO-HCFA vs CA | 1.65 (0.68,4.00) p=0.27 | 1.83 (0.93,3.62) p=0.08 | 1.10 (0.49,5.46) p=0.82 | 0.87 (0.40,1.91) p=0.73 |
| HO-HCFA vs CA | 2.51 (1.01,6.28) p=0.05 | 1.31 (0.62,2.75) p=0.48 | 0.97 (0.41,2.27) p=0.94 | 0.86 (0.39,1.90) p=0.71 |
| Severity score (per unit higher) | N/A | N/A | N/A | 1.30 (0.87,1.92) p=0.20 |
| Ribotype 027 | 0.97 (0.47,2.03) p=0.94 | 1.39 (0.69,2.81) p=0.36 | 177.2 (58.4,537.6) p<0.001 | 102.0 (41.0,253.4) p<0.001 |
| Ribotype 015 | 0.85 (0.35,2.07) p=0.72 | 1.25 (0.59,2.65) p=0.56 | 2.13 (0.96,4.74) p=0.06 | 2.29 (1.03,5.08) p=0.04 |
| Ribotype 078 | 0.93 (0.40,2.15) p=0.87 | 1.73 (0.80,3.74) p=0.16 | 6.63 (3.24,13.5) p<0.001 | 7.19 (3.52,10.7) p<0.001 |
| Ribotype 014 | 0.47 (0.17,1.28) p=0.14 | 0.72 (0.29,1.76) p=0.47 | 3.42 (1.58,7.41) p=0.002 | 5.03 (2.36,10.5) p<0.001 |
| Ribotype 020 | 0.99 (0.39,2.48) p=0.98 | 2.34 (1.05,5.20) p=0.04 | 3.87 (1.64,9.15) p=0.002 | 4.47 (1.90,5.42) p=0.001 |
| Ribotype 002 | 1.15 (0.53,2.49) p=0.73 | 1.03 (0.47,2.26) p=0.94 | 2.01 (0.92,4.40) p=0.08 | 2.52 (1.17,12.1) p=0.02 |
| Ribotype 001/072 | 1.23 (0.43,3.48) p=0.70 | 1.76 (0.70,4.43) p=0.23 | 5.61 (2.24,14.1) p<0.001 | 4.79 (1.90,2.58) p=0.001 |
| Other ribotype | 1.00 | 1.00 | 1.00 | 1.00 |
| Having multiple positive samples | N/A | N/A | N/A | 1.53 (0.91,2.58) p=0.11 |
| Having a previous donor 0-2 SNVs vs >10 SNVs | 1.61 (0.87,3.00) p=0.13 | 1.62 (0.91,2.88) p=0.10 | N/A | N/A |
| Having a previous donor 0-2 SNV vs 3-10 SNVs vs >10 SNVs | 1.57 (0.83,2.96) p=0.17 | 0.96 (0.53,1.76) p=0.90 | N/A | N/A |

* Included in final model with the best fitting transform the inverse of months from study start, with lower odds of identifying a previous 0-2SNV donor lower only in the first month of the study (OR=0.42 per 1/(months from start of study) higher (95% CI 0.20,0.87) p=0.02).
Note: OR=odds ratio. Included factors are those in Tables 3 and 4 (main text). N/A means this factor not applicable for this model (either is the outcome, occurs after the outcome or is a competing outcome).

**Figure S5: Genetic relatedness of new CDI cases (n=640) to any previous isolate based on origin of recipient’s infection.**

**Table S3: Genetic relatedness of new CDI cases to any previous isolate based on origin of recipient’s infection.**

| **Match to any previous case** | **Community** | **Indeterminate** | **Community onset hospital associated** | **Hospital onset hospital associated** | **p-value** |
| --- | --- | --- | --- | --- | --- |
|  | (CA)  (N=121) | (I)  (N=46) | (CO-HCFA)  (N=126) | (HO-HCFA)  (N=347) |  |
| 0 SNV | 14 (12%) | 9 (20%) | 32 (25%) | 107 (31%) | <0.001 |
| 0-2 SNV | 26 (21%) | 11 (24%) | 42 (33%) | 148 (43%) | <0.001 |
| 0-2 SNV match and direct ward contact | 0 (0%) | 7 (15%) | 22 (17%) | 86 (25%) | 0.12 |
| 0-2 SNV match and spore contact only | 0 (0%) | 2 (4%) | 6 (5%) | 22 (6%) | 0.74 |
| 0-10 SNV | 54 (45%) | 21 (46%) | 71 (56%) | 208 (60%) | 0.02 |
| Ribotype match | 106 (88%) | 39 (85%) | 114 (90%) | 308 (89%) | 0.75 |

Note: numbers show n (%)

**Figure S6. (a) Ribotype diversity and (b) Simpson’s index of diversity by origin of infection**

(a)

(b)


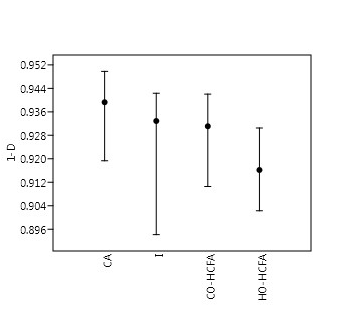


**Table S4: Genetic relatedness to previous CDI cases by ribotype (if >25 cases)**

| **Ribotype** | **Frequency** | **0-10 SNV** | **0-2 SNV** | **0 SNV** | **0-2 SNVs** | **0-2 SNVs** |
| --- | --- | --- | --- | --- | --- | --- |
|  |  |  |  |  | (Direct ward contact) | (No contact) |
| **027** | 105 | 102 (97) | 99 (94) | 85 (81) | 67 (64) | 10 (10) |
| **015** | 62 | 35 (56) | 12 (19) | 9 (15) | 2 (3) | 9 (15) |
| **078** | 57 | 54 (95) | 26 (46) | 7 (12) | 6 (11) | 14 (25) |
| **002** | 55 | 28 (51) | 15 (27) | 10 (18) | 8 (15) | 6 (11) |
| **014** | 49 | 20 (41) | 18 (37) | 13 (27) | 10 (20) | 6 (12) |
| **020** | 38 | 25 (66) | 14 (37) | 8 (21) | 5 (13) | 5 (13) |
| **001** | 29 | 17 (59) | 13 (45) | 10 (34) | 7 (24) | 3 (10) |
| **Other** | 245 | 73 (30) | 30 (12) | 20 (8) | 10 (4) | 16 (7) |

Note: figures show N (row percentage) for individual ribotypes with >25 cases.

**Table S5a Antibiotic use in** **genetically-matched and distinct recipients**

| **Antibiotic use^a^** | **All new cases** | **New cases with previous 0-2 SNV donor** | **New cases with previous 3-10 SNV donor** | **New cases with all prior samples >10 SNVs** | **P-value**  **0-2 vs 3-10 vs >10 SNVs** | **P-value**  **0-2 vs >2 SNVs** | |
| --- | --- | --- | --- | --- | --- | --- | --- |
|  | (N=215) | (N=99) | (N=43) | (N=73) |  |  | |
|  |  |  |  |  |  | |  |
| - Fluoroquinolone received in the last 90 days | 25/215 (12%) | 12/99 (12%) | 7/43 (16%) | 6/73 (8%) | p=0.42 | | p=0.84 |
| - Number of antibiotic agents in the last 90 days | 3 (2-3) [0-8] | 3 (2-4) [0-7] | 3 (2-4) [1-8] | 3 (1-3) [0-8] | p=0.61 | | p=0.79 |
| - >2 agents in the last 90 days | 112/215 (52%) | 50/99 (51%) | 24/43 (56%) | 38/73 (52%) | p=0.84 | | p=0.67 |
| - Any antibiotics received in the last 7 days | 139/215 (65%) | 66/99 (67%) | 33/43 (77%) | 40/73 (55%) | p=0.05 | | p=0.57 |
| - Number of antibiotic agents in the last 7 days | 1 (0-2) [0-4] | 1 (0-2) [0-3] | 1 (1-2) [0-4] | 1 (0-1) [0-4] | p=0.04 | | p=0.44 |
| - >2 agents in the last 7 days | 22/215 (10%) | 8/99 (8%) | 8/43 (19%) | 6/73 (8%) | p=0.13 | | p=0.34 |

^a^ Applicable only to hospital-onset cases: collected from January 2011 and where data was available.

**Table S5b Antibiotic use in** **genetically-matched and distinct potential donors**

| **Antibiotic use^a^** | **All new cases** | **0-2 SNV potential donors to any subsequent case^b^** | **Subsequent case 3 to 10 SNV** | **All subsequent cases >10 SNV** | **P-value**  **0-2 vs 3-10 vs >10 SNVs** | **P value**  **0-2 vs >10 SNVs** | |
| --- | --- | --- | --- | --- | --- | --- | --- |
|  | (N=215) | (N=85) | (N=35) | (N=95) |  |  | |
| - Fluoroquinolone received in the last 90 days | 25/215 (12%) | 8/85 (9%) | 7/35 (20%) | 10/95 (11%) | p=0.23 | | p=0.41 |
| - Number of antibiotic agents in the last 90 days | 3 (2-3) [0-8] | 3 (2-4) [0-8] | 3 (2-4) [1-8] | 3 (2-3) [0-8] | p=0.60 | | p=0.70 |
| - >2 agents in the last 90 days | 112/215 (52%) | 44/85 (52%) | 20/35 (57%) | 48/95 (51%) | p=0.80 | | p=0.94 |
| - Any antibiotics received in the last 7 days | 139/215 (65%) | 55/85 (65%) | 27/35 (77%) | 57/95 (60%) | p=0.19 | | p=0.99 |
| - Number of antibiotic agents in the last 7 days | 1 (0-2) [0-4] | 1 (0-2) [0-3] | 1 (1-2) [0-3] | 1 (0-1) [0-4] | p=0.14 | | P=0.66 |
| - >2 agents in the last 7 days | 22/215 (10%) | 11/85 (13%) | 4/35 (11%) | 7/95 (7%) | p=0.45 | | p=0.29 |

^a^ Applicable only to hospital-onset cases: collected from January 2011 and where data was available.

**References**

1. Lunter G, Goodson M. Stampy: A statistical algorithm for sensitive and fast mapping of Illumina sequence reads. Genome Res 2011;21:936–9.
2. Li H, Handsaker B, Wysoker A, et al. The Sequence Alignment/Map format and SAMtools. Bioinformatics 2009;25:2078–9.
3. Guindon S, Dufayard J-F, Lefort V, Anisimova M, Hordijk W, Gascuel O. New algorithms and methods to estimate maximum-likelihood phylogenies: assessing the performance of PhyML 3.0. Syst Biol 2010;59:307–21.
4. Cohen SH, Gerding DN, Johnson S, et al. Clinical practice guidelines for Clostridium difficile infection in adults: 2010 update by the Society for Healthcare Epidemiology of America (SHEA) and the Infectious Diseases Society of America (IDSA). *Infection Control* 2010;31(05):431-455
